# Mucus trail tracking in a predatory snail: olfactory processing retooled to serve a novel sensory modality

**DOI:** 10.1002/brb3.198

**Published:** 2013-12-18

**Authors:** Kinjal Patel, Nagma Shaheen, Jessica Witherspoon, Natallia Robinson, Melissa A Harrington

**Affiliations:** 1 Department of Biology, Delaware State University1200 North Dupont Highway, Dover, Delaware, 19901; 2 Department of Physical Therapy and Rehabilitation Service, University of Kansas Medical CenterKansas City, Kansas, 66160

**Keywords:** *Euglandina*, invertebrate model, multielectrode array, olfaction, sensory learning, trail following

## Abstract

**Introduction:**

The rosy wolfsnail (*Euglandina rosea*), a predatory land snail, finds prey snails and potential mates by following their mucus trails. *Euglandina* have evolved unique, mobile lip extensions that detect mucus and aid in following trails. Currently, little is known of the neural substrates of the trail-following behavior.

**Methods:**

To investigate the neural correlates of trail following we used tract-tracing experiments in which nerves were backfilled with either nickel-lysine or Lucifer yellow, extracellular recording of spiking neurons in snail procerebra using a multielectrode array, and behavioral assays of trail following and movement toward the source of a conditioned odor.

**Results:**

The tract-tracing experiments demonstrate that in *Euglandina*, the nerves carrying mucus signals innervate the same region of the central ganglia as the olfactory nerves, while the electrophysiology studies show that mucus stimulation of the sensory epithelium on the lip extensions alters the frequency and pattern of neural activity in the procerebrum in a manner similar to odor stimulation of the olfactory epithelium on the optic tentacles of another land snail species, *Cantareus aspersa* (previously known as *Helix aspersa*). While *Euglandina* learn to follow trails of novel chemicals that they contact with their lip extensions in one to three trials, these snails proved remarkably resistant to associative learning in the olfactory modality. Even after seven to nine pairings of odorant molecules with food, they showed no orientation toward the conditioned odor. This is in marked contrast to *Cantareus* snails, which reliably oriented toward conditioned odors after two to three trials.

**Conclusions:**

The apparent inability of *Euglandina* to learn to associate food with odors and use odor cues to drive behavior suggests that the capability for sophisticated neural processing of nonvolatile mucus cues detected by the lip extensions has evolved at the expense of processing of odorant molecules detected by the olfactory system.

## Introduction

Chemical senses are arguably the oldest and most important sensory modalities in the animal kingdom. The earliest animals on the planet most likely navigated their environments by responding to chemical cues, and even now animals of all phyla rely on some type of chemo-sensation to obtain food, avoid predators, and find mates. Land snails and slugs are highly sensitive to odors and display robust associative conditioning to olfactory cues (Gelperin 1975; Kemenes 1989; Alkon and Nelson 1990; Sahley et al. 1990; Sahley and Crow 1998; Balaban 2002). These characteristics combined with their simple and relatively accessible nervous systems make them useful model systems for studying the neural substrates of sensory processing and learning, particularly in the chemosensory modality.

In many species of snails and slugs, the receptor cells of the olfactory epithelia (located on the two optical tentacles) send axons through olfactory nerves to a pair of cerebral ganglia (Hubendick 1955). Electrophysiological and imaging analyses have demonstrated that olfactory information processing and olfactory learning in many species of slugs and snails occurs in the procerebrum located at the point where the olfactory nerve joins the cerebral ganglion (Chase 1985; Gelperin and Tank 1990; Kimura et al. 1998; Straub et al. 2004; Ierusalimsky and Balaban 2010). The procerebrum consists of a layer of small, densely packed cell bodies and two separate layers of neuropil. The procerebrum shares several characteristics with the olfactory bulb of mammals, including large, spontaneous oscillations in the local field potential (Delaney et al. 1994) that are changed in frequency and amplitude by odor stimulation (Gelperin and Tank 1990; Gervais et al. 1996; Gelperin 1999). Work with the slug, *Limax maximus*, has shown that odor-cued associative conditioning alters the activity of procerebral neurons in a spatially specific way (Kimura et al. 1998; Teyke et al. 2000). Given the small size of the nervous systems of snails and slugs: ∼80,000–100,000 cells, approximately 75% of which are in the procerebra (Gelperin and Tank 1990; Balaban 2002), it is likely that the procerebrum plays a critical role in sensory processing in general, not just olfactory processing. Investigating a snail model in which a sensory modality other than olfaction is a significant determinant of behavior can shed light on the extent that the procerebrum is involved in processing of information in other sensory modalities.

Snails, similar to other gastropods, secrete mucus from their foot which aids in locomotion, acting as both glue and a lubricant (Denny 1980a,b1980b). The mucus is left behind by the animal, forming a trail. Many species of gastropod have been reported to follow mucus trails of their own and other species to find mates, return to a “home” location, and in some cases to catch prey (for review see (Wells and Buckley 1972; Ng et al. 2013).

*Euglandina rosea*, the rosy wolfsnail, is a carnivorous land snail native to the Southeastern U.S. It tracks down its prey (other snails and slugs) as well as potential mates by following the mucus trails they leave behind. *Euglandina* snails follow mucus trails using a sophisticated chemosensory system that is separate from olfactory sensing (Chiu and Chou 1962). Previous work has shown that the sensory epithelia adapted for detecting mucus are on the long, mobile lip extensions that are absent in other snail species (Cook 1985a,b1985b; Clifford et al. 2003). While tracking prey, the *Euglandina* constantly touch their lip extensions to the trail being followed. In laboratory experiments, they do not appear to detect mucus trails at a distance, and amputation of the optic (olfactory) tentacles had no effect on trail following while amputation of the lip extensions caused a large deficit in trail following (Cook 1985b; Clifford et al. 2003). The dependence of *Euglandina* on their lip extensions for mucus trail following is particularly striking given that other snails and slugs are able to follow trails of odors or mucus using their optic tentacles (Chase and Croll 1981; Cook 1985c).

In the field, *Euglandina* are voracious predators that, except for a specific, possibly distasteful slug, are known to eat almost any molluscan prey they encounter (Cook 1985b, 1989; Kinzie 1992; Gerlach 1999, 2001; Meyer and Cowie 2010; Davis-Berg 2011). In the laboratory, *Euglandina* easily distinguish mucus of prey snails from that of other *Euglandina*. Although mucus trails from other *Euglandina* are followed at approximately the same frequency as prey snails (∼90% of all trails encountered) adult *Euglandina* rarely attack other *Euglandina*. Similarly, prey snails that have been covered with *Euglandina* mucus are usually ignored after a brief inspection, while *Euglandina* that have been covered with prey mucus are rapidly attacked by the predator snails (Shaheen et al. 2005). *Euglandina* also show robust chemosensory learning. They will follow artificial trails of novel, nonvolatile chemicals after only one or two trials of eating a prey snail coated with the chemical, but they do not learn to follow the artificial trails if exposure to test compounds is not paired with feeding on a prey snail (Clifford et al. 2003). Not only are *Euglandina* able to learn to follow artificial trails associated with food they also learn to follow trails of novel chemicals that have been paired with exposure to a conspecific (Shaheen et al. 2005). These results show that, in the mucus sensing modality, *Euglandina* have a sophisticated associative learning ability in which both food and access to potential mates can act as a reward to reinforce a voluntary behavior (following a trail of a novel compound). While previous work has demonstrated the centrality of mucus sensing to *Euglandina* behavior, it is not known how neural processing of mucus stimuli is carried out in the central ganglia. In addition, while the presence of odorants has been shown to disrupt trail following (Clifford et al. 2003) very little is known about the role of olfactory sensing in driving the voluntary behavior of *Euglandina*.

In this study, we sought to identify the neural pathways and processing that are important for mucus trail chemosensation in *Euglandina* and compare them to those involved in odor processing in a similarly sized, herbivorous land snail species, *Cantareus aspersa*. While there has been a report of trail following by *Cantareus* snails, trail following is not a prominent part of their behavioral repertoire. This is in sharp contrast to *Euglandina*, who follow nearly all trails encountered regardless of their state of hunger or satiety, and even if characteristics of the snail that they are following make it unsuitable for consumption (Cook 1985b, 1989; Kinzie 1992; Gerlach 2001; Clifford et al. 2003; Davis-Berg 2011).

We also compared the relative significance of odor and mucus cues in directing *Euglandina* behavior by attempting to train the animals to orient toward food-associated odorants, and comparing their learning performance with that of *Cantareus* snails exposed to identical training paradigms.

## Methods

### Anatomy

Tract-tracing experiments with *Cantareus* and *Euglandina* nerves using nickel-lysine or Lucifer yellow as back-filling dyes were done according to the methods of (Fredman 1987; Hernadi 2000). *Euglandina* and *Cantareus* snails were anesthetized by injecting 1–3 mL of cold 50 mmol/L MgCl_2_ into the neck. The central ganglia connected to the optical and oral tentacles, and the lip extensions were dissected out of the snail, and the nerve coming from the sensory epithelia was cut and sucked into a micropipette containing a solution of 10 mmol/L nickel-lysine. The nerves were left in the nickel-lysine overnight at 4°C; then the snail brain was developed for one half hour with 3 mmol/L rubeanic acid, fixed overnight with 2% paraformaldehyde, dried with an alcohol series, and then cleared with methyl salicylate (wintergreen oil). In staining the superior tentacle nerves of both *Cantareus* and *Euglandina*, the optical nerve was separated from the olfactory nerve, and only the olfactory nerve was sucked into the pipette with the nickel-lysine. In staining the lip extension nerve of *Euglandina*, the nerve was cut between the cerebral ganglia and the joining of the lip extension nerve and inferior tentacle.

For backfilling experiments with Lucifer yellow, we followed the same procedure except that the backfilled brains were not developed, but simply fixed and dried with the alcohol series before visualizing with a fluorescent microscope (Olympus IX-71).

*Euglandina* central ganglia were stained with toluidine blue by modifying the procedures of (Altman 1980). Snail brains were dissected out of the snail and mounted on slides using Meyer's albumin fixative. After staining, the tissue was dehydrated and cleared with methyl benzoate.

### Electrophysiology

Local field potential (LFP) oscillations were recorded from *Cantareus* and *Euglandina* procerebra using the Panasonic MED64 multielectrode recording system (Automate Scientific, Berkeley, CA). The MED64 probes contain 64 electrodes in an 8 × 8 matrix with interelectrode spacing of 75 *μ*m. The electrodes are embedded in the center of a transparent glass dish.

*Euglandina* and *Cantareus* snails were anesthetized by injecting 1–3 mL of cold 50 mmol/L MgCl_2_ into the neck. A single procerebrum connected to the superior and inferior tentacle nerves (*Cantareus*), and the lip extension nerves (*Euglandina*) was dissected out of the snail. The skin of the optical or lip extensions that contain the sensory epithelium was left intact and attached to the nerves. The ganglion was laid across the electrode grid and pressed onto the grid with a slab of 2% agarose with the nerves and sensory epithelium uncovered by the agarose. To assess the effect of sensory stimulation, dilute solutions of odorant or mucus in water were applied to the sensory epithelia and the response of the neural networks was measured. Electrode traces were sampled at 20 kHz and the data were preprocessed by applying IIR Butterworth filters to remove 60 Hz power interference harmonics. High-frequency components (>5 kHz) that do not correspond to biological processes were removed using FIR LF filter with linear phase.

### Paired association procedure

Each snail was tested for a baseline attraction to a dilute solution of each odorant before any other exposure to the odorant. After the initial test, each *Euglandina* was fed a prey snail (juvenile *Cantareus*, their regular diet in the lab) and 1–2 drops of a dilute odorant solution were dropped onto its radula as it ate. Because the procerebra were laid whole across the electrodes, the electrodes recorded neural activity from superficial cells in the cell mass layer.

Dilute solutions of four naturally occurring odorants were used. We chose 10% solutions of cinnamon oil, almond oil, bay oil, and anise oil as these are complex mixtures with multiple volatile compounds, and since they are used in food were likely to be safe for the snails to eat. A different odorant solution was used for each behavioral experiment so that the odor would be novel in the baseline condition. The snails were housed in a different room from where the feeding trials took place, which was also different from the room in which the test trials were run. The radula is the tooth-lined tongue that snails use to draw food into their mouths. *Cantareus* snails were fed minced carrots as their regular diet in the lab, and for the experiments, 1–2 drops of the dilute odorant were dropped on their radulas as they ate the carrot. The snails were tested again for attraction to the odorant 24–48 h after each training session in which eating was paired with exposure to an odorant.

### Tests for formation of olfactory associations

The ability of *Euglandina* and *Cantareus* to learn to approach a novel odor through association of the odor with food was tested using three methods. In the first method, a cotton swab soaked in odorant (a 10% solution of either cinnamon oil or almond oil) was placed at the upper left corner of a 21 × 27.5 cm transparency sheet. The test snail was placed in the lower right corner of the same sheet facing the swab and at least 20 cm away from it. The snails were allowed to crawl until they left the transparency sheet. The mucus trails of the snails were visualized by sprinkling the sheets with charcoal powder and rinsing under running water. The snail's sticky mucus trails trapped the dark powder so it remained on the sheet as the rest of the powder was washed away (Karowe et al. 1993). The distance from the snail's trail to the swab was measured and the closest point on the sheet was recorded. If the snails left the transparency sheet without moving toward the odorant, the closest distance to the swab was the starting point—20 cm away from the swab. Data from all snails tested were included in the analysis, regardless of whether they initially moved toward the swab or away from it. Significance of the data was tested with an ANOVA.

**Figure 1:**
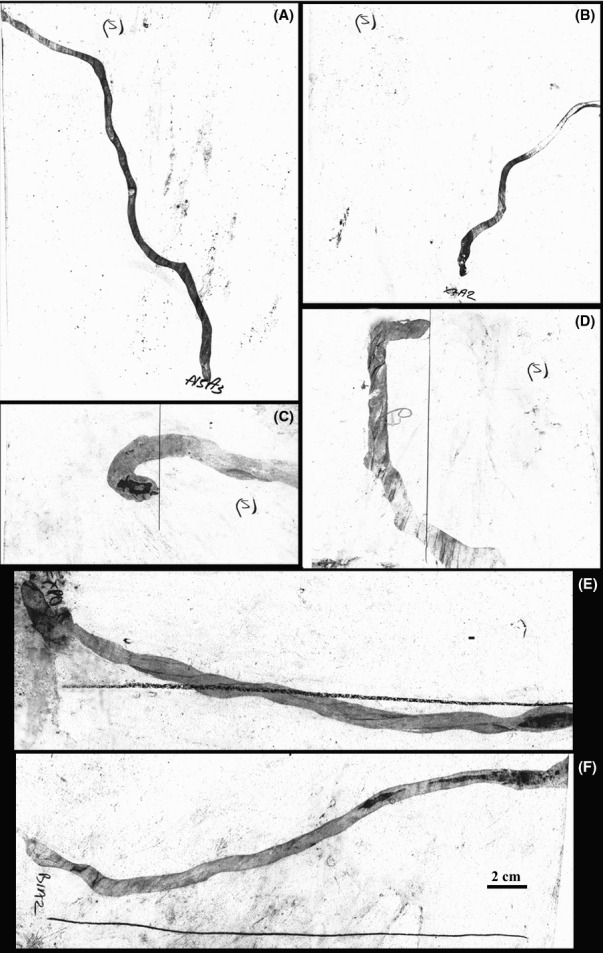
Sample trails left by test *Euglandina* during odor learning experiments. The movements of the snails are tracked by visualizing the mucus trails with charcoal. (A, B) Odorant-soaked cotton swabs—location marked with (s)—were placed at one end of a transparency sheet and test snails were placed at the other end. In A, the snail approached very close to the swab, while in B the snail moved opposite to the swab. (C, D) An odorant-soaked swab—location marked with (s)—was placed behind the snails. In C, the snail turned to approach the swab, and the trial was scored as “attracted.” In D, the snail did not turn to approach the swab within 10 cm of the initial location so the trial was scored as “not attracted.” (E, F), Following an artificial trail of odorant (at location of pen line). Snails initial location at left corner of the sheet. In E, the test snail was scored as following the trail. In F, the test snail was scored as not following. The pen lines to mark the location of the swabs, the odorant trails and the “attracted/not attracted” line were all added after the conclusion of the behavioral experiment.

For the previous experiment, the snails were placed facing the odorant, and so might have a bias to move toward it that would affect the results. To ensure that the direction the snails faced was not the deciding factor in the decision to move toward the odor, we used a different approach to measure the attractiveness of the test odor. In the second type of odor learning experiment, a cotton swab soaked in a different odorant (10% bay oil) was placed in the middle of a 21 × 27.5 cm transparency sheet. The test *Euglandina* or *Cantareus* snail was placed 10 cm from the swab and facing the opposite direction. The test snails were allowed to crawl until they left the transparency sheet, and the trails were visualized with charcoal powder. Experiments were scored “attracted” versus “not attracted” based on whether the test snail turned around and moved toward the swab. Snails that turned around and traveled toward the swab past the point where the back of their shell had been placed at the start were scored as “attracted.” To be scored as “attracted” the snails had to travel back past the point there they were originally placed within about three body lengths (∼10 cm) distance from that point. Snails which did not turn around or did not travel past the point where they were placed at the start of the experiment within 10 cm were scored as “not attracted” (see Fig. 1C and D for examples). Significance of the data was tested with Logistic regression.

The ability of *Euglandina* to learn to follow artificial trails of an odorant chemical was tested by painting a streak of 10% anise oil on a transparency sheet, placing the snail 5 cm away from the chemical trail and allowing it to crawl across it. After the experiment, a marker pen was used to mark where the odorant trail was laid and the movement of the snail was visualized by sprinkling the sheet with charcoal powder and rinsing off the excess. After the first test of following the artificial trail, the snail was fed a prey snail while the anise solution was dropped on its radula, and the snails were tested for following of the trail again in 24–48 h. Snails were judged to have followed the trail if their mucus trail was superimposed over or paralleled the anise trail for at least three body lengths (approximately 10 cm). Significance was tested with a Logistic regression. For examples of typical olfactory and trail-following olfactory association experiments see Figure 1.

All behavior tests were conducted in conditions that were as consistent as possible. The experiments were performed under artificial lighting in the same windowless room. The testing arenas (the transparency sheets) were arranged on the same bench and in the same orientation for every trial. Snails from both species were oriented in the same direction relative to the room at the start of each trial for all of the behavioral tests.

## Materials

All chemicals were purchased from Sigma-Aldrich Chemicals (St Louis, MO).

## Results

### Neuroanatomy

**Figure 2:**
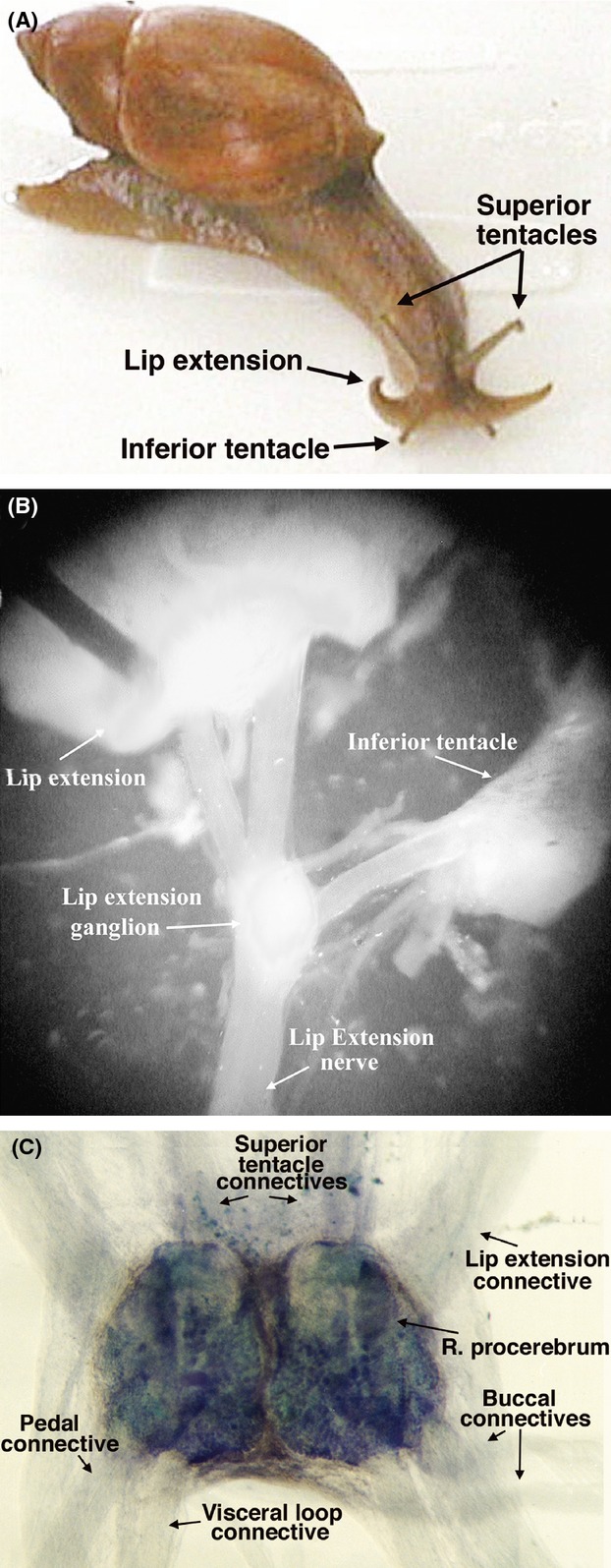
Morphology and gross anatomy of the *Euglandina*, the lip extension nerve and cerebral ganglia (A) *Euglandina rosea*. The superior tentacles are also known as the optic tentacles, the inferior tentacles are also known as the oral tentacles. (B) Dissection of the *Euglandina* lip extension and neck showing the joining of the nerves from the lip extension and the inferior (oral) tentacle. (C) Toluidine blue staining of *Euglandina* cerebral ganglia. The small, densely packed cell bodies of the procerebrum are visible.

*Euglandina* follow the mucus trails of prey and conspecific snails using the lip extensions visible in Figure 2A. As shown in Figure 2B, two large nerves connect the lip extension to a ganglion in the neck of the snail, and this ganglion is also connected to the oral tentacle nerve. A single large nerve, separate from the olfactory nerve, connects this “lip extension ganglion” to the cerebral ganglion, which enters laterally on the procerebrum while the olfactory nerve enters anteriorally (see Fig. 2C).

**Figure 3:**
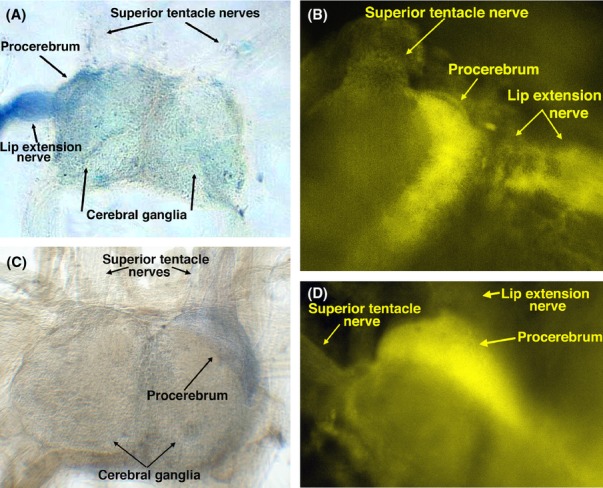
Backfilling of either lip extension nerve or superior tentacle nerve labels the procerebrum. (A) Cerebral ganglia of a *Euglandina* with the lip extension nerve backfilled with nickel-lysine. (B) Cerebral ganglia of a different *Euglandina* with the lip extension nerve backfilled with Lucifer yellow. These figures are representative of a total of 5 similar experiments with different *Euglandina*. (C) Cerebral ganglia of a *Euglandina* with the superior tentacle nerve backfilled with nickel-lysine. Representative of two similar experiments. (D) Cerebral ganglion of a *Euglandina* with the superior tentacle nerve backfilled with Lucifer yellow. Representative of three similar experiments.

Backfilling experiments were done by dissecting the olfactory or lip extension nerve from the tentacle, cutting the end and dipping it into a solution of either nickel-lysine or the fluorescent dye, Lucifer yellow. Developing the nickel-lysine back-filled brain with rubeanic acid gives a blue color where the nickel accumulated, while fluorescent imaging reveals the accumulation of Lucifer yellow. Figure 3 shows the results of two different backfilling experiments in which the lip extension nerve of a *Euglandina* was backfilled with nickel-lysine or Lucifer yellow (Fig. 3A and B). Both dyes show an accumulation in the procerebrum. In the Lucifer yellow backfill, the higher contrast possible shows that the labeling is in the crescent shape that is typical of the cell body layer of the procerebrum. As sensory nerves project from the periphery to the center, these backfilling experiments indicate that neurons carried in the lip extension nerve synapse in the procerebrum of the cerebral ganglion. Figure 3 also shows results of two backfilling experiments in which the olfactory nerves of two different *Euglandina* were backfilled with nickel-lysine (Fig. 3C and D). The blue accumulated in the procerebrum just as with experiments in which the lip extension was backfilled.

**Figure 4:**
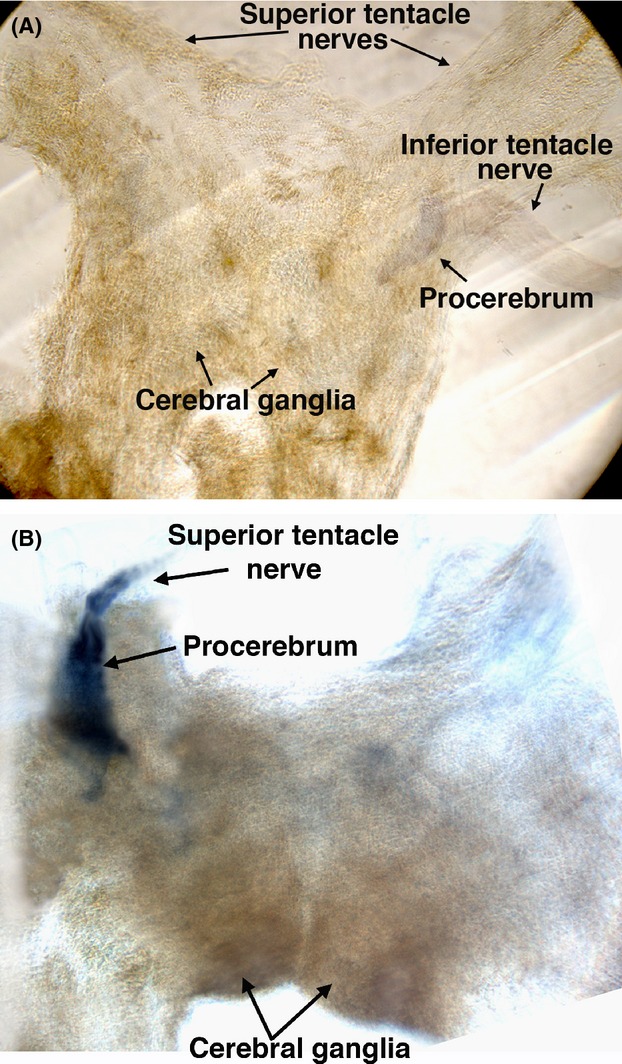
Backfilling of nerves for superior and oral tentacles in *Cantareus* snails also labels the procerebrum. (A) Cerebral ganglia from a *Cantareus* snail with the inferior tentacle nerve backfilled with nickel-lysine. Representative of two similar experiments. (B) Cerebral ganglia from a *Cantareus* snail with the superior tentacle nerve backfilled with nickel-lysine. Representative of three similar experiments.

Similar backfilling experiments were performed with *Cantareus* snails. Both the optical and oral tentacles were backfilled with nickel-lysine. As shown by the deposition of nickel from the backfilling, in *Cantareus*, the oral tentacle nerve enters laterally on the cerebral ganglia and innervates the procerebrum (Fig. 4A). When olfactory nerves of *Cantareus* are backfilled, deposits of nickel and Lucifer yellow appear in the procerebrum as well, but cover a larger area than the labeling when the inferior tentacle is backfilled (Fig. 4B). The crescent shape of the labeling of the procerebra of both *Euglandina* and *Cantareus* is consistent with the shape of the cell body layer in the procerebrum (Nagy and Sakharov 1970; Ermentrout et al. 1998) suggesting that neurons in the optical, oral, and lip extension nerves synapse in the cell body layer of the procerebrum.

### Electrophysiology

**Figure 5:**
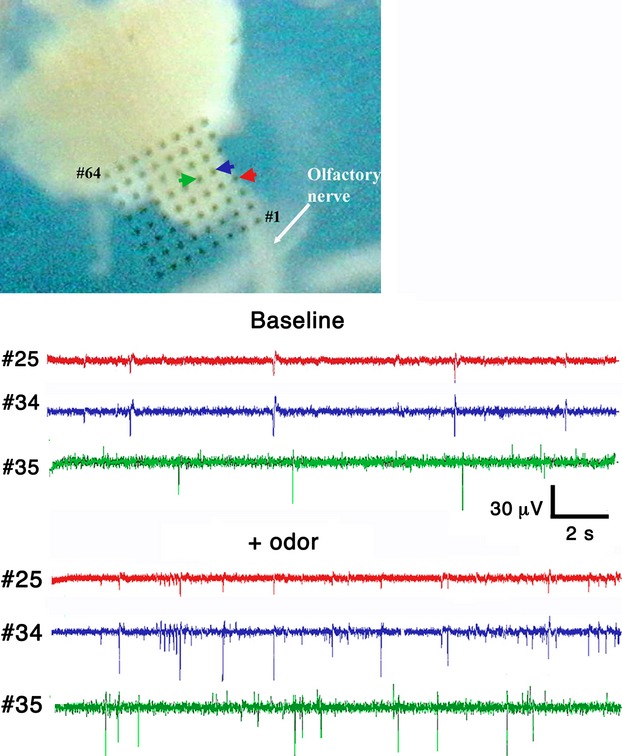
Multielectrode recordings from a *Cantareus aspersa* procerebrum show oscillatory activity that is activated by odor stimulation. Top: Image of *Cantareus* snail ganglia on electrode array with displayed electrodes identified with arrows. Lower panel: Spike activity recorded simultaneously on several electrodes (electrode number as indicated). Colors correspond to the particular electrode trace shown. The frequency of spike activity is significantly increased in response to odorant molecules (bay oil) applied to the olfactory epithelium on the optical tentacle.

Oscillations in the local field potential (LFP) that change in frequency and amplitude in response to odor stimulation have been recorded from the cerebral ganglia in a number of mollusks including the slug*, Limax maximus* (Gelperin and Tank 1990) and the snail *Helix pomatia* (Chase 1981; Pin and Gola 1987; Schütt et al. 1999). As shown in Figure 5, separate electrodes of the MED64 are able to record oscillations from *Cantareus* ganglia that are increased in frequency by the application of an odorant (10% bay oil) to the sensory epithelium of the tentacle. Interestingly, electrodes at the lateral edge of the procerebrum (#25 and #34) record a different pattern of LFP oscillations than an electrode placed more medially, and maintain a separate rhythm even after odor stimulation. Fifteen active electrodes were recorded from the cerebral ganglia of four different snails. Average spike frequency was 0.32 ± 0.04 Hz before odorant application and 1.48 ± 0.31 Hz after (*P *<* *0.05; Kruskal–Wallis test).

**Figure 6:**
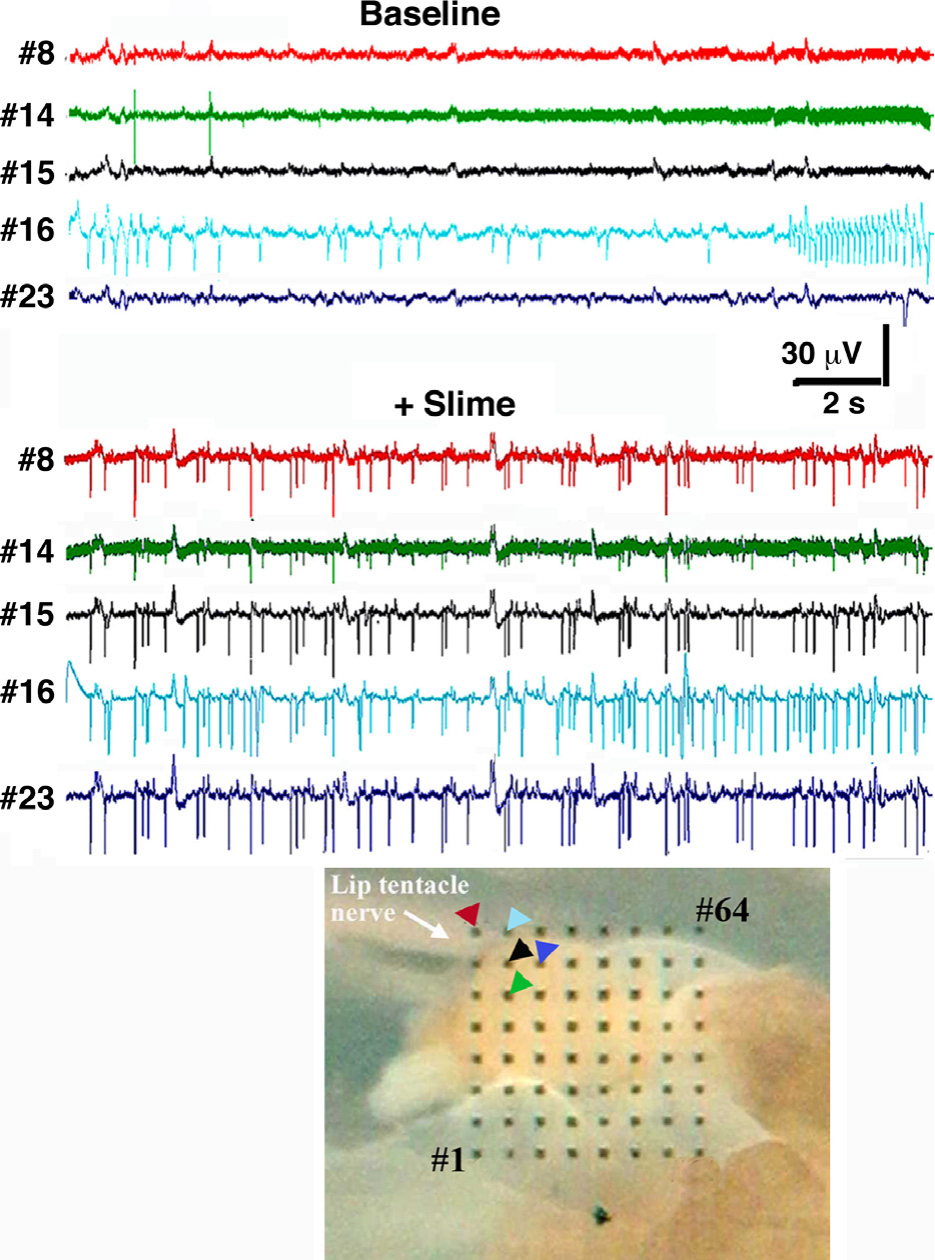
Multielectrode recordings from a *Euglandina procerebrum* show that neuronal spiking is activated by mucus stimulation of lip extension. Sample traces showing spike activity recorded simultaneously from five electrodes from a *Euglandina* ganglia. Notice the increase in the baseline noise over the time of the baseline recording from electrode #14. Bottom Panel: Image of *Euglandina* snail ganglia on electrode array with displayed electrodes identified with arrows. Colors correspond to the particular electrode trace shown.

Similarly, recordings from *Euglandina* ganglia (Fig. 6) show an increase in both frequency and amplitude of LFP oscillations after stimulation of the lip extension epithelium with a mucus solution. As with *Cantareus* ganglia, the pattern of the oscillation varies in different parts of the procerebrum. Notice that before mucus stimulation, each electrode has a slightly different pattern of activity, even the electrodes closest together (numbers 14–16). After mucus stimulation an oscillating activity of frequency 3–8 Hz develops. The oscillation is largely synchronized across all electrodes, although some electrodes miss some peaks of the oscillations. Thirteen active electrodes were recorded from the cerebral ganglia of two different snails. Average spike frequency was 0.81 ± 0.53 Hz before odorant application and 2.84 ± 0.55 Hz after (*P *<* *0.05; Kruskal–Wallis test).

**Figure 7:**
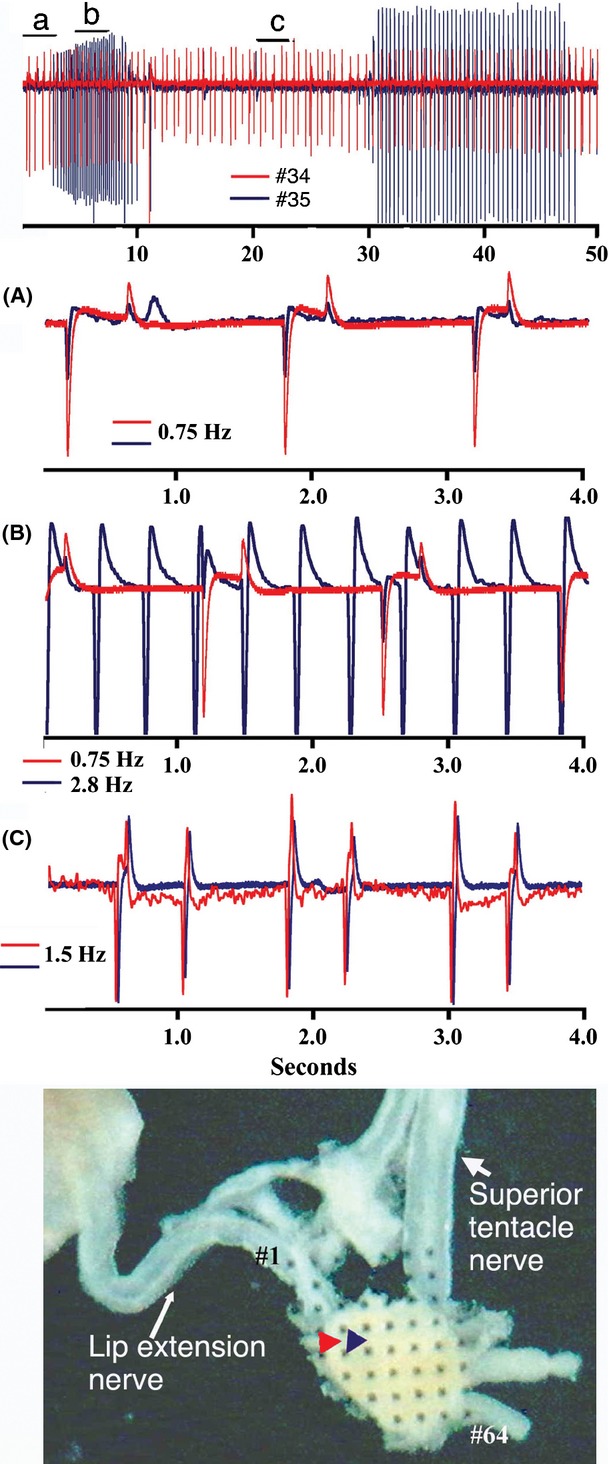
Close neighbor spiking neurons in mucus-stimulated ganglia fall in and out of synchronization. Sample traces showing spike activity recorded simultaneously from two different electrodes from a *Euglandina* ganglia stimulated by mucus applied to lip extension. Traces labeled a, b, and c are 4 second expanded samples of the regions under the bars in the top panel. Bottom Panel: Image of *Euglandina* snail ganglia on electrode array with displayed electrodes identified with arrows. Colors correspond to the particular electrode trace shown.

Interestingly, in *Euglandina* ganglia that were stimulated by mucus applied to the lip extension, the neural activity recorded by neighboring electrodes alternated between periods of synchronization and desynchronization (Fig. 7). Even when the activity recorded by neighboring electrodes followed different rhythms, there was frequently a regular pattern of spikes that were synchronized (e.g., every third or fourth spike).

### Odor conditioned behavior

**Figure 8:**
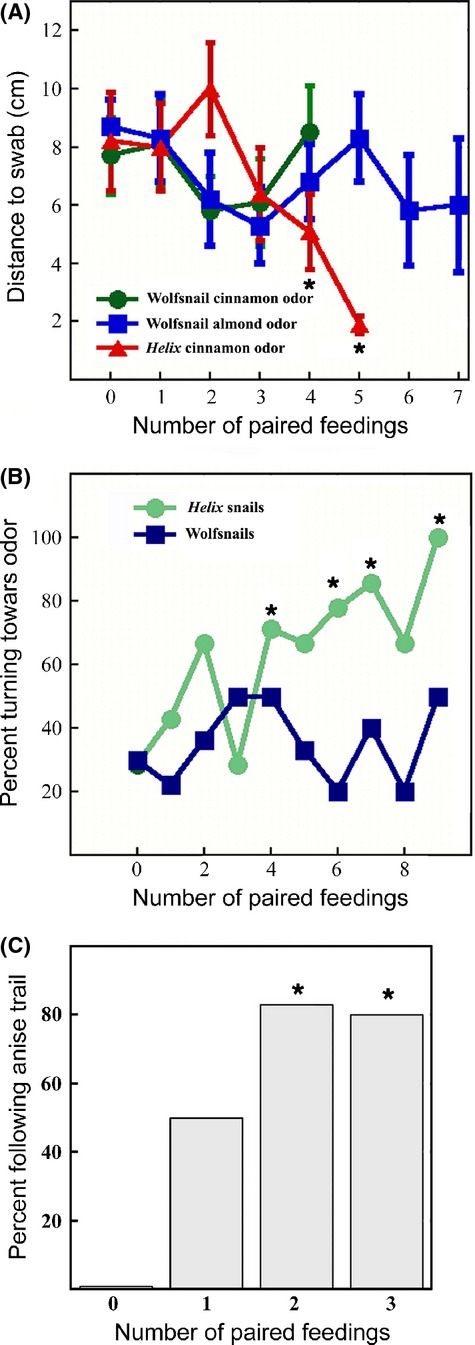
*Euglandina* appear capable of minimal olfactory learning compared to *Cantareus* snails. The 0 time points represent baseline trials in which the snails had no prior exposure to the odorant. (A) Results from conditioning experiment where the distance from snail trail to an odorant-soaked swab was measured at the closest point. Seven *Euglandina* were tested for learning for each of two different odors, while seven *Cantareus* were tested on a single odor. Data are means ± SEM. Significance was tested by ANOVA. Points at which the average distance to the swab was significantly different than the baseline at the *P *<* *0.05 level are indicated with *. (B) Percent of snails turning toward odor on a swab was measured with nine *Cantareus* snails and 10 *Euglandina*. Significance was tested with a logistic regression. For the significant data points (indicated by *) the following values were obtained: four paired feedings χ^2^ = 4.22, *P *=* *0.040; six paired feedings χ^2^ = 5.12, *P *=* *0.023; seven paired feedings χ^2^ = 4.89, *P *=* *0.027; nine paired feedings χ^2^ = 4.24, *P *=* *0.039. (C) Seven Euglandina were tested for following of an artificial trail of an odorant (bay oil). The 0 time point represents baseline with no prior exposure to bay oil. Significance was tested with logistic regression, and is indicated with *. For the significant data points, the following values were obtained: two paired feedings χ^2^ = 4.77, *P *=* *0.029; three paired feedings χ^2^ = 4.98, *P *=* *0.026.

*Cantareus* snails and *Euglandina* showed remarkable differences in their response to having a novel odor paired with consumption of food. *Euglandina* were tested with three different odor association paradigms using dilute solutions of three different complex, naturally occurring odorants. Compared to *Cantareus* snails*, Euglandina* were markedly less efficient at learning to approach conditioned odors. In the first test for odor association, snails were assessed for changes in their approach to a cotton swab containing an odorant that had been paired with food. In the baseline trial, prior to any feeding exposure, both *Cantareus* and *Euglandina*, on the average approached within 7–9 cm of the swab before leaving the sheet. After several paired feedings, the *Cantareus* snails came much closer to the swab, averaging only 2 cm away by the fifth trial. In contrast, with two different odorants, the average closest distance that *Euglandina* approached the swab did not change, even after seven paired feedings (Fig. 8A).

In a second odor-learning test with a different odorant, *Cantareus* and *Euglandina* were placed facing away from a swab coated with the odorant, allowed to crawl, and the direction that they crawled was scored as “attracted” if they turned around to crawl toward the swab and “not attracted” if they did not turn around. As with the distance to swab test, the *Cantareus* snails performed much better. In the baseline test, about 30% of the snails (*Cantareus* or *Euglandina*) turned toward the odorant. After the second paired feeding, more than 50% of the *Cantareus* snails turned around and moved toward the odorant, and after eight paired feedings, 100% of the *Cantareus* test snails turned around to approach the odorant. In contrast, the *Euglandina*'s performance never got above chance. At best, only 50% of the *Euglandina* snails turned toward the odor (after nine paired feedings), and there was no trend with increasing numbers of paired feedings (Fig. 8B).

The apparent inability of *Euglandina* to learn to travel toward novel odors associated with food is in marked contrast to their ability to learn to follow artificial trails of novel chemicals. Previous experiments with nonvolatile compounds showed that *Euglandina* learn to follow novel trails after one to three paired feedings (Clifford et al. 2003), and can learn to follow artificial trails paired with exposure to a potential mate as well as exposure to food (Shaheen et al. 2005). To rule out the possibility that the *Euglandina's* poor learning performance might be due to an inability to detect the volatile compounds that were used, we tested their ability to learn to follow an artificial trail of a new odorant molecule. After a baseline trial with an artificial trail of 10% anise oil, we fed test *Euglandina* prey snails paired with a solution of 10% anise oil. Twenty-four hours later, the snails were placed near an artificial trail of dilute anise oil and observed for trail following. Similar to what we have previously observed with nonvolatile artificial trails (Clifford et al. 2003), after a single paired feeding 50% of the test snails followed the artificial trail, with 80% of them following after two paired feedings.

## Discussion

Laboratory experiments with the predatory snail *Euglandina* have shown that these snails have a highly developed ability to detect mucus from other snails and slugs and to select a response to mucus cues from a repertoire of several behaviors. Previous work has shown that based on cues in mucus, *Euglandina* can distinguish between prey snails and conspecifics as well as favored and unfavored prey species (Cook 1989; Clifford et al. 2003; Meyer and Cowie 2010) reacting differently to mucus trails depending on the identity of the trail layer. In the laboratory, the snails can tell the directionality only of conspecific trials, apparently by distinguishing the right side of the trail from the left (Shaheen et al. 2005), while in the wild, the snails appear to be able to determine trail directionality from prey trails as well (Davis-Berg 2011). *Euglandina* also rapidly learn to follow trails of novel chemicals associated with either prey snails or potential mates (Shaheen et al. 2005). While it is not certain that snail mucus does not contain volatile compounds that could contribute to trail attractiveness, previous work has shown that mucus trail following does not require olfaction, as amputating the optical tentacles has little impact on mucus trail following, while amputating the lip extensions eliminates trail following in most *Euglandina* tested (Cook 1985a; Clifford et al. 2003).

While the olfactory systems and olfactory learning abilities of several species of slugs and snails have been extensively studied (Chase 1981, 1985; Chase and Tolloczko 1993; Gelperin 1994; Gervais et al. 1996; Sahley and Crow 1998; Balaban 2002), almost nothing is known about the anatomy and physiology of mucus trail chemosensation. This study identifies connections between the lip extensions that mediate mucus trail detection and the cerebral ganglia, and demonstrates that mucus stimuli detected by the lip extensions are processed in the same central ganglia and in the same manner as odor molecules detected by the olfactory system. Our anatomical and tract-tracing experiments show that in the *Euglandina*, the nerve from the inferior tentacle joins with the nerve from the lip extension, and the combined nerve connects to the procerebral lobe where neurons from the lip extension synapse in the cell body layer. While a large swelling at the point where the lip extension nerve and oral tentacle nerve comes together suggests a ganglion, it is unlikely that afferent nerves from the sensory epithelium terminate at this point as nickel-lysine and Lucifer yellow taken up by the distal ends of lip extension nerve are transported past this point to the cerebral ganglion. Our results suggest that the connectivity and processing of input from the lip extension may have arisen in the *Euglandina* as an elaboration of the neural processing dedicated to the oral tentacle in other snails and slugs. This hypothesis is supported by our observation that in *Euglandina*, the lip extension nerve and oral tentacle nerve join, and the joined nerve enters the cerebral ganglion in the mid-lateral area where the oral tentacle enters in other land snails.

In the *Euglandina*, backfilling the lip extension nerve produces extensive labeling of the procerebrum appearing to cover the entire procerebrum, and resembles the results of backfilling of the *Cantareus* olfactory nerve more than the *Cantareus* oral tentacle nerve backfilling. Labeling of the *Euglandina* olfactory nerve produces labeling of the procerebrum that looks substantially the same as the labeling produced by backfilling the lip extension nerve.

In addition to the similarity between the anatomical labeling, the neuronal activity of the *Euglandina* procerebrum is similar to neuronal activity recorded from the procerebra of other land snails. The activity is characterized by a widespread oscillation in local field potential with a frequency of 0.1–0.3 Hz, and stimulation with odorants changes the frequency and amplitude of the oscillations (Chase 1981; Gelperin and Tank 1990; Kimura et al. 1993; Delaney et al. 1994; Ermentrout et al. 1998). Multielectrode recording from *Euglandina* ganglia shows large increases in the frequency of the oscillations after mucus was applied to the lip extension, and an increase in the synchronization of the activity across a wide area of the procerebrum. Similar results were obtained with multielectrode recordings from *Cantareus* ganglia, where stimulating with an odorant on the olfactory epithelium increased the frequency of the synchronized field potential oscillation across a large stretch of the procerebrum.

Most intriguingly, our results demonstrate that *Euglandina* have paid a price for their highly developed responsiveness to mucus. *Euglandina* are very efficient at learning to follow trails of novel compounds associated with eating a prey snail or contact with a potential mate, as long as they can contact the compounds with their lip extensions (Clifford et al. 2003; Shaheen et al. 2005). However, they are strikingly ineffective at learning to orient or move toward novel odors detectable only with the olfactory sense on their optic tentacles, even when those odors have been repeatedly associated with food. Their lack of ability to learn that an odor is associated with a food source is in striking contrast to the abilities of *Cantareus aspersa*, another land snail of similar size, which learns to move toward a conditioned odor in just a few trials. The *Euglandina*'s lack of ability to learn from odors is unlikely to be due to an inability to detect them, as earlier results have demonstrated that the presence of a strong odor can disrupt mucus trail following (Cook 1985a; Clifford et al. 2003). While not all of the specific odors tested in this study are in the native range of *Euglandina*, studies of olfaction in numerous species support the hypothesis that odor detection and olfactory transduction involve basic mechanisms that are universal across most species in most phyla (Hildebrand and Shepherd 1997,) so it is very unlikely that *Cantareus* snails could detect these odors while *Euglandina* individuals could not. Moreover, *Euglandina* are as efficient in learning to follow trails of volatile compounds as they are with nonvolatile compounds, once they are able to touch the trail with their lip extensions. This suggests that it is route of detection that is crucial, not the specific odors being tested.

Another possibility is that the dilute solutions of cinnamon, almond, and bay oils that we used as odorants are somehow aversive to *Euglandina*, and that prevents them from approaching the odors even when associated with food. Even if that is the case, similar studies with *Limax maximus*, have demonstrated appetitive conditioning to odors that were initially aversive to the slugs (Sahley and Crow 1998), suggesting that initial aversion can be overcome by pairing an odor with food.

The *Euglandina* has developed sophisticated central mechanisms to process mucus cues and use them to drive its behavior, and our data show that this seems to have occurred at the expense of processing of olfactory cues using the olfactory epithelium on the optic tentacles.

Our learning experiments demonstrate that, unlike other land snails and slugs, *Euglandina* appear to draw only limited information from odor stimuli, and the central processing capability devoted to odors does not appear to be enough to support associative learning. Given that land snails have only ∼40,000 neurons in each procerebrum (Gelperin and Tank 1990; Balaban 2002), the limited processing power available may have forced this trade-off during the evolution of the trail-following behavior.
